# Hepcidin regulation in Kenyan children with severe malaria and non-typhoidal *Salmonella* bacteremia

**DOI:** 10.3324/haematol.2021.279316

**Published:** 2022-07-01

**Authors:** Kelvin M. Abuga, John Muthii Muriuki, Sophie M. Uyoga, Kennedy Mwai, Johnstone Makale, Reagan M. Mogire, Alex W. Macharia, Shebe Mohammed, Esther Muthumbi, Salim Mwarumba, Neema Mturi, Philip Bejon, J. Anthony G. Scott, Manfred Nairz, Thomas N. Williams, Sarah H. Atkinson

**Affiliations:** 1 Kenya Medical Research Institute (KEMRI) Center for Geographic Medicine Research, KEMRI-Wellcome Trust Research Programme, Kilifi, Kenya; 2 Department of Public Health, School of Human and Health Sciences, Pwani University, Kilifi, Kenya; 3 Epidemiology and Biostatistics Division, School of Public Health, University of the Witwatersrand, South Africa; 4 Open University, KEMRI-Wellcome Trust Research Programme – Accredited Research Centre, Kilifi, Kenya; 5 Centre for Tropical Medicine and Global Health, Nuffield Department of Clinical Medicine, University of Oxford, Oxford, UK; 6 Department of Infectious Disease Epidemiology, London School of Hygiene and Tropical Medicine, London, UK; 7 Department of Internal Medicine II, Medical University Innsbruck, Innsbruck, Austria; 8 Department of Infectious Diseases and Institute of Global Health Innovation, Imperial College, London, UK; 9 Department of Paediatrics, University of Oxford, Oxford, UK

**Keywords:** Hepcidin, iron, malaria, non-typhoidal *Salmonella*, children, anemia, Africa

## Abstract

Malaria and invasive non-typhoidal *Salmonella* (NTS) are life-threatening infections that often co-exist in African children. The iron-regulatory hormone hepcidin is highly upregulated during malaria and controls the availability of iron, a critical nutrient for bacterial growth. We investigated the relationship between *Plasmodium falciparum* malaria and NTS bacteremia in all pediatric admissions aged ≤5 years between August 1998 and October 2019 (n=75,034). We then assayed hepcidin and measures of iron status in five groups: (1) children with concomitant severe malarial anemia (SMA) and NTS (SMA+NTS, n=16); and in matched children with (2) SMA (n=33); (3) NTS (n=33); (4) cerebral malaria (CM, n=34); and (5) community-based children. SMA and severe anemia without malaria were associated with a two-fold or more increased risk of NTS bacteremia, while other malaria phenotypes were not associated with increased NTS risk. Children with SMA had lower hepcidin/ferritin ratios (0.10 [IQR 0.03, 0.19]) than those with CM (0.24 [0.14, 0.69]; P=0.006) or asymptomatic malaria (0.19 [0.09, 0.46]; P=0.01) indicating suppressed hepcidin levels. Children with SMA+NTS had lower hepcidin levels (9.3 ng/mL [4.7, 49.8]) and hepcidin/ferritin ratios (0.03 [0.01, 0.22]) than those with NTS alone (105.8 ng/mL [17.3, 233.3]; P=0.02 and 0.31 [0.06, 0.66]; P=0.007, respectively). Since hepcidin degrades ferroportin on the *Salmonella*-containing vacuole (SCV), we hypothesize that reduced hepcidin in children with SMA might contribute to NTS growth by modulating iron availability for bacterial growth. Further studies are needed to understand how the hepcidin-ferroportin axis might mediate susceptibility to NTS in severely anemic children.

## Introduction

Malaria and invasive non-typhoidal *Salmonella* (NTS) are major causes of illness and death among children living in sub-Saharan Africa. According to the World Health Organization (WHO), 94% of the 409,000 malaria-associated deaths in 2019 occurred in the sub-Saharan African region, with children under five years of age being disproportionately vulnerable.^1^ In this region, NTS bacteremia is also common accounting for 80% of the estimated 535,000 global cases in 2017.^2^ While NTS is commonly associated with self-limiting gastroenteritis in European populations, NTS infections in African children can cause life-threatening sepsis with case fatality rates of 20-25%.^2, 3^ NTS bacteremia is highly prevalent in areas with concurrent malaria endemicity,^4-6^ and reduced malaria incidence has been associated with a decrease in NTS bacteremia.^7, 8^ The association between NTS and malaria has been particularly observed in children with severe malarial anemia (SMA),^5, 9-11^ but this has not been reported in all settings.^12, 13^

**Figure 1:**
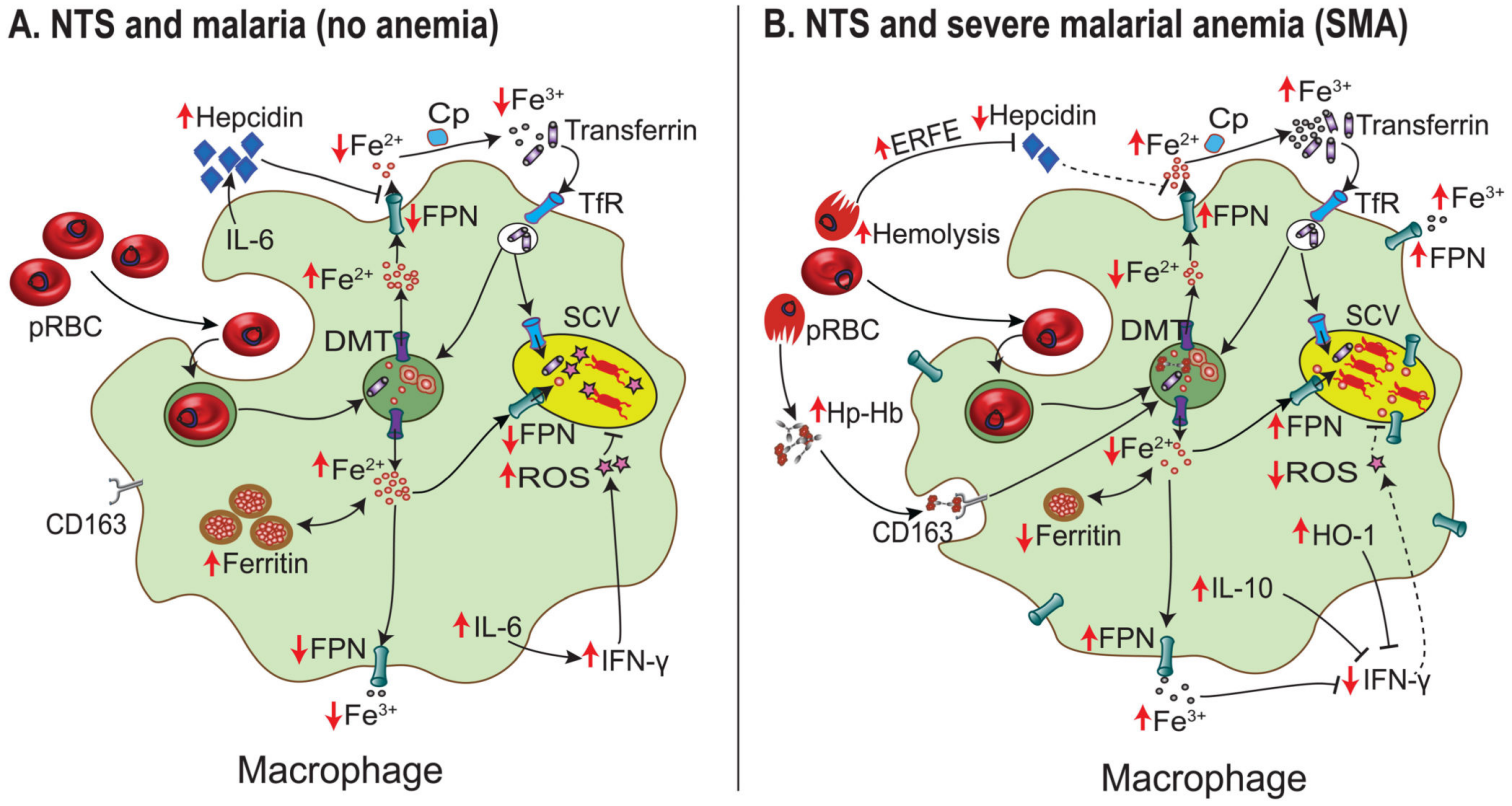
The hepcidin-link between severe malarial anemia (SMA) and non-typhoidal *Salmonella* (NTS) bacteremia. Low hepcidin levels in children with SMA may contribute to the risk of NTS bacteremia. (A) During malaria infection, proinflammatory responses and parasitemia induce the expression of hepcidin, the main iron regulatory hormone. Hepcidin degrades ferroportin (FPN) on the macrophage membrane and the *Salmonella* containing vacuole (SCV),^21^ resulting in decreased iron availability for NTS bacteria. The bacteria may also utilize other iron acquisition strategies such as transferrin through transferrin receptors (TfR) in early endosomes. Proinflammatory responses, including production of interleukin (IL)-6 and interferon-gamma (IFN-γ), mediate killing of NTS through formation of reactive oxygen species (ROS) and other pathways. (B) In SMA, increased hemolysis and erythropoietic drive induce production of erythroferrone (ERFE),^36^ a hormone that downregulates hepcidin synthesis. This results in increased expression of FPN on the surface of the macrophage and the SCV.^21^ Heme from hemolyzed parasitized red blood cells (pRBC) and the haptoglobin-hemoglobin (Hp-Hb) complex is broken down by heme oxygenase-1 (HO-1) into equimolar amounts of iron, biliverdin and carbon monoxide. HO-1 and heme-breakdown products downregulate immune responses and promote an anti-inflammatory environment.^15^ The net effect of low hepcidin, increased HO-1, SMA-induced anti-inflammatory cytokines such as IL-10 and increased intra-SCV iron levels is increased proliferation of NTS bacteria. DMT-1 denotes divalent metal transporter 1; and Cp, ceruloplasmin. Red arrows indicate direction of increase or decrease; dotted lines indicate reduced activity.

SMA may increase susceptibility to NTS bacteremia via a number of contributory pathways including sustained hemolysis, accumulation of free heme from lysed red blood cells, increased gut permeability, disruption of immune responses, and upregulation of heme oxygenase-1 (Figure 1).^14^ Heme oxygenase-1 impairs neutrophil oxidative burst capacity, reduces neutrophil bactericidal activity, and promotes iron accumulation in macrophages.^15, 16^ Recent *in vitro* and animal studies suggest that hepcidin, the master iron regulator^17^, may also play an important role in determining susceptibility to NTS by controlling the availability of iron,^18-20^ a nutrient critical for bacterial growth and proliferation.^16, 19^ Hepcidin degrades ferroportin, the sole iron exporter, which was recently shown to transport iron across the *Salmonella*-containing vacuole (SCV).^21, 22^ In murine studies, low hepcidin levels and increased ferroportin expression on the SCV are associated with increased susceptibility to *Salmonella* Typhimurium infections (Figure 1).^18, 22^ However, there are no studies of hepcidin in NTS infection in humans. In this study, we investigated the relationship between malaria, anemia and NTS in 75,034 hospitalized Kenyan children over a 21-year period and then estimated levels of hepcidin and other iron biomarkers in children with NTS bacteremia and malaria.

## Methods

### Study design and participants

**Figure 2:**
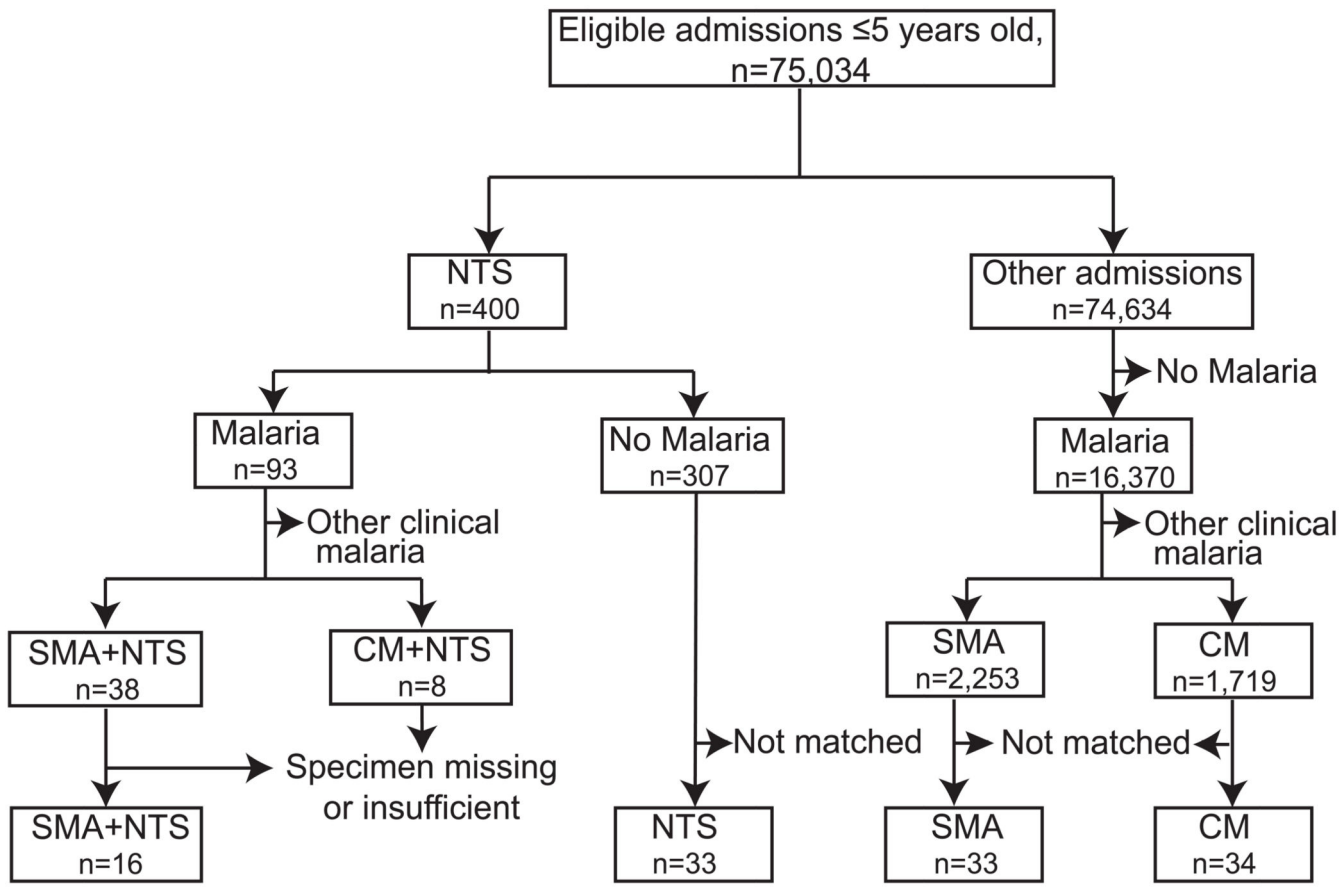
Selection of study participants. All children aged ≤60 months with complete age and hemoglobin data admitted between August 1998 and October 2019 were included in the retrospective epidemiological analysis. Children with concomitant severe malaria and non-typhoidal *Salmonella* (NTS), and whose specimens were available in the Kilifi biobank, were enrolled into the iron and hepcidin sub-study. Each child was then matched with two hospitalized children with NTS alone, severe malaria anemia (SMA) alone, and cerebral malaria (CM) alone based on age and sex.

Ethical approval was granted by the Scientific Ethics Review Unit of the Kenya Medical Research Institute and informed consent was provided by parents or guardians (Supplementary Methods). The study was conducted in Kilifi, a rural malaria-endemic area along the Kenyan coast. The estimated incidence rate of NTS bacteremia among children <5 years was 36.6 cases/100,000 person-years between 1998 to 2014.^23^ Our study included two parts:

1. We investigated the relationship between malaria and NTS bacteremia among all pediatric admissions (n=75,034) aged ≤60 months admitted between 1^st^ August 1998 and 31^st^ October 2019 with complete age, malaria and hemoglobin data.
2. We then measured hepcidin, iron and inflammatory markers from five groups of children including those hospitalized with: 1) SMA and NTS coinfection (SMA+NTS); 2) SMA alone; 3) NTS alone; and 4) cerebral malaria (CM) and 5) community-based children with and without asymptomatic malaria using stored biobank samples over the 21-year time period. Each child from group 1 was matched with two from each of the other hospitalized groups based on age and sex (Figure 2). The community-based children were part of an ongoing birth cohort evaluating malaria immunity,^24^ and their samples were selected from a single cross-sectional bleed in May 2002.

### Laboratory procedures

Thick and thin blood films were stained with Giemsa and examined for *Plasmodium falciparum* using standard methods. Samples for bacterial culture were collected in BACTEC^®^ Peds Plus bottles and processed with a BACTEC-8050 automated blood-culture instrument (Becton-Dickson, UK). Positive samples were sub-cultured and serological tests and biochemical test kits (API, bioMérieux) were used to confirm suspected pathogens.^23, 25^ *Bacillus* species, *Micrococcus* species, viridans group *Streptococcus*, coagulase negative *Staphylococcus*, and coryneforms were considered contaminants. Rapid antibody tests were used for HIV-1 testing. Sickle cell disease was diagnosed using polymerase chain reaction (Supplementary Methods). Iron and inflammatory biomarkers were assayed as previously described^26^ (Supplementary Methods).

### Clinical definitions

For children with *Plasmodium falciparum* malaria, we defined SMA as hemoglobin <5 g/dl and CM as Blantyre coma score <3 according to WHO criteria.^27^ Severe anemia was defined as hemoglobin <5 g/dl; fever as temperature >37.5□C; and NTS bacteremia as isolation of *Salmonella enterica* subspecies excluding Typhi or Paratyphi serovars in blood cultures.

### Statistical analyses

All data were analyzed using STATA 15.1 (StatCorp. College Station, Texas, USA). For all pediatric admissions, we used univariable and multivariable logistic regression models to investigate for putative risk factors for NTS bacteremia. We used a causal directed acyclic graph to assess the suitability of covariates for multivariable analyses (Supplementary Figure S1), and a stepwise backward selection regression method to fit the final multivariable models (Supplementary Methods). We also analyzed the relationship between SMA and risk of other bacterial organisms. In the hepcidin sub-study, continuous data were reported as medians and interquartile ranges (IQR) and compared using the Wilcoxon rank-sum test. Non-parametric Spearman’s correlation evaluated associations between variables. We normalized nonnormally-distributed variables by loge-transformation and used multivariable linear regression models to adjust for inflammation (C-reactive protein) and year of admission.

## Results

### Study of all hospital admissions

A total of 75,034 children aged ≤60 months were admitted to Kilifi County Hospital during the 21-year study period and had complete data for analysis. Median age was 11.8 months (IQR 2.2, 26.1) and 42,450 (56.6%) were male. *P*. *falciparum* malaria was identified in the blood films of 16,463 (21.9%) hospitalized children; of whom 2,291 (13.9%) had SMA, 1,727 (10.5%) had CM, and 416 (2.5%) had concomitant SMA and CM. Pathogenic bacterial organisms were isolated from 3,792 (5.1%) blood cultures. NTS bacteremia was identified in 400 (10.5%) of the positive blood cultures. Of the NTS isolates, 309 were serotyped and 45.0% (139/309) were *Salmonella enterica* serovar Enteritidis and 44.3% (137/309) were serovar Typhimurium, while 10.7% (33/309) were not typeable. The prevalence of NTS bacteremia has decreased over the years as malaria has also decreased (Supplementary Figure S2).

NTS bacteremia was identified in 93/16,463 (0.6%) hospitalized children with *P*. *falciparum* malaria, including 38/2,291 (1.7%) with SMA and 8/1,727 (0.5%) with CM. SMA was associated with a two-fold increased risk of NTS bacteremia in the final multivariable model (adj. OR 2.17 [95% CI 1.44, 3.28]; P=0.0002, Supplementary Table S2). However, a positive malaria slide (OR 1.08 [95% CI 0.85, 1.36]; P=0.52) and CM (OR 1.00 [95% CI 0.50, 2.02]; P=0.99) were not associated with increased risk of NTS bacteremia (Table 1). Children with malaria but without SMA had a 39% reduced risk of NTS bacteremia (OR 0.61 [95% CI 0.45, 0.82]; P=0.001, Table 1). Children with severe anemia without malaria parasitemia also had an increased risk of NTS bacteremia in final multivariable models (adj. OR 4.03 [95% CI 2.78, 5.84]; P<0.0001, Supplementary Table S2). The risk of NTS bacteremia increased by 26% for each 1 g/dL decrease in hemoglobin levels in all children (adj. OR 1.26 [95% CI 1.21, 1.32]; P<0.0001) and by 51% in children with malaria parasitemia (adj. OR 1.51 [95% CI 1.36, 1.68]; P<0.0001). In final multivariable models, other significant risk factors for NTS bacteremia were younger age, fever, diarrhea, sickle cell disease, very severe pneumonia, underweight and, in restricted models, HIV status (Supplementary Table S2). SMA was not associated with increased risk of other bacterial organisms causing bacteremia (Supplementary Figure S3).

### Hepcidin sub-study

We included 116 hospitalized children in the following groups: 1) 16 with SMA+NTS; 2) 33 with SMA alone; 3) 33 with NTS alone; and 4) 34 with CM (Figure 2); and 5) 291 community-based children with (n=49) and without (n=242) asymptomatic malaria parasitemia. The clinical characteristics of children in the sub-study are shown in Supplementary Table S3.

### Hepcidin levels in children with malaria

**Figure 3:**
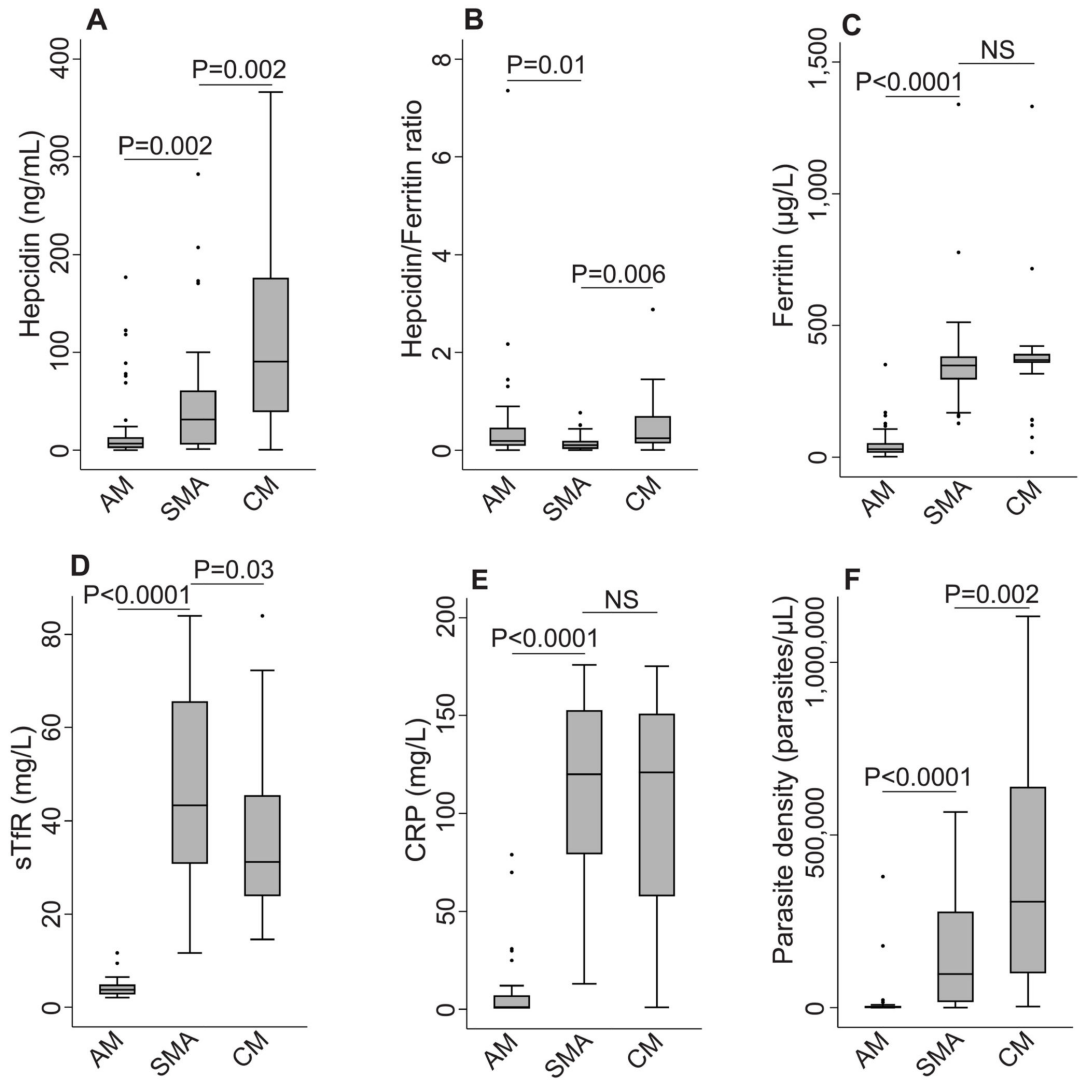
Iron and inflammatory biomarkers in children with malaria. Circulating levels of (A) hepcidin, (B) ferritin, (C) hepcidin/ferritin ratio, (D) soluble transferrin receptors (sTfR), (E) C-reactive protein (CRP), and (F) hemoglobin in children with malaria. P values from pairwise comparisons were determined by the Wilcoxon rank-sum test. ‘NS’ indicates P > 0.05. AM, asymptomatic malaria; CM, cerebral malaria; SMA, severe malaria anemia.

We first compared hepcidin levels among children with malaria. Hepcidin levels were lower in children with SMA (median 31.1 ng/ml [IQR 5.5, 61.2]) compared to those with CM (90.7 ng/ml [IQR 38.7, 176.1]; P=0.002). However, both of these severe malaria groups had higher hepcidin levels than children with asymptomatic malaria parasitemia living in the community (Figure 3A). We found similar hepcidin levels in community-based children with and without asymptomatic malaria parasitemia (6.5 ng/ml [IQR 2.0, 13.1] and 3.8 ng/ml [IQR 1.2, 12.6], respectively). Hepcidin expression was suppressed in children with SMA as evidenced by a lower hepcidin/ferritin ratio (0.10 [IQR 0.03, 0.19]) compared to those with CM (0.24 [IQR 0.14, 0.69]; P=0.006), or asymptomatic parasitemia (0.19 [IQR 0.09, 0.46]; P=0.01; Figure 3B).

We then explored differences in putative regulators of hepcidin. Children with SMA had increased erythropoietic drive as indicated by higher sTfR levels (43.3 mg/L [IQR 30.8, 65.6]) than those with CM (31.2 mg/L [IQR 23.9, 45.5]; P=0.03), although ferritin and CRP levels did not differ between the groups (Figure 3). Hospitalized children had higher levels of ferritin, sTfR, and CRP and higher *P*. *falciparum* parasite densities than those living in the community (Figure 3 C-F).

### Hepcidin levels in children with malaria and NTS

**Figure 4:**
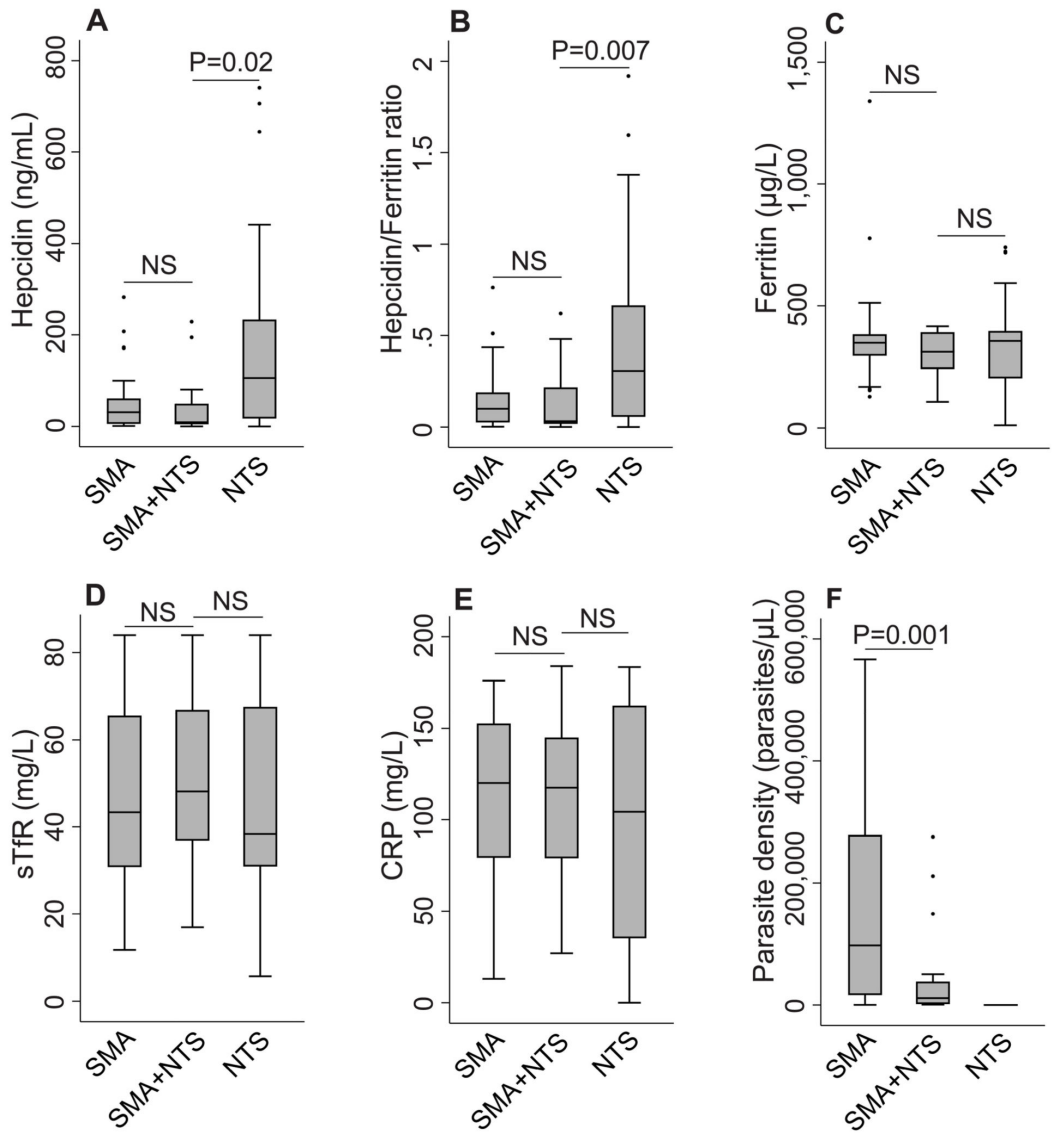
Iron and inflammatory biomarkers in children with severe malaria anemia (SMA) and/or non-typhoidal *Salmonella* (NTS) bacteremia. Circulating levels of (A) hepcidin, (B) ferritin, (C) hepcidin/ferritin ratio, (D) soluble transferrin receptors (sTfR), (E) C-reactive protein (CRP), and (F) hemoglobin in children with SMA and/or NTS bacteremia. P values from pairwise comparisons were determined by the Wilcoxon rank-sum test. ‘NS’ indicates P > 0.05.

We then considered hepcidin levels in children with malaria and NTS. Hepcidin levels were lower in children with SMA+NTS (9.3 ng/ml [IQR 4.7, 49.8]) compared to those with NTS alone (105.8 ng/ml [IQR 17.3, 233.3]; Figure 4A, Table 2). Hepcidin/ferritin ratios were also lower in children with SMA+NTS (0.03 [IQR 0.01, 0.22]) compared to those with NTS alone (0.31 [IQR 0.06, 0.66]; P=0.007; Table 2, Figure 4B). In a linear regression model controlled for CRP and year of admission, hepcidin levels were two-fold higher in children with NTS compared to those with SMA+NTS (adj. β 1.99 [95% CI 0.81, 3.26]; P=0.001, Supplementary Table S4), although sTfR, ferritin, and CRP levels did not differ between the groups (Figure 4 C-E). Only one participant in the sub-study had sickle cell disease and excluding the participant from the analysis did not alter our findings.

Hepcidin levels were positively correlated with ferritin (r=0.38, P=0.0001), CRP (r=0.31, P=0.0007), hemoglobin (r=0.37, P<0.0001) and parasite density (r=0.44, P<0.0001), and negatively correlated with sTfR (r=-0.37, P<0.0001) among the hospitalized children.The direction and strength of correlation between hepcidin and its predictors varied across individual groups as shown in Supplementary Table S5.

## Discussion

Malaria and NTS are important causes of hospitalization and death among children living in sub-Saharan Africa.^1, 2^ In this study, we analyzed retrospective data from 75,034 hospitalized children aged ≤60 months and found that SMA, but not CM or other malaria phenotypes, was associated with increased risk of NTS bacteremia. Children with severe anemia of all causes, both with or without malaria parasitemia, also had an increased risk of NTS bacteremia. In a sub-study investigating iron biomarkers, children with SMA had lower hepcidin levels than children with CM. We also found that children with SMA+NTS had lower hepcidin levels than children with NTS alone. We did not find differences in ferritin or CRP levels among hospitalized children, but children with SMA alone and SMA+NTS had lower hepcidin/ferritin ratios and higher sTfR levels. Children living in the community with asymptomatic parasitemia had lower levels of hepcidin, ferritin, CRP and sTfR and lower parasite densities than hospitalized children.

Children with SMA had a two-fold increased risk of NTS bacteremia compared to those without SMA. This risk was not observed in children with CM or other malaria phenotypes that excluded severe anemia. Moreover, each 1g/dl decrease in hemoglobin concentrations in children with malaria was associated with a 51% increase in the risk of NTS bacteremia. SMA increased the risk of NTS, but not other bacteria suggesting an NTS-specific effect rather than a generalized immunological failure to control bacteremia (Supplementary Figure S3). Previous studies across sub-Saharan Africa have also reported an increased risk of NTS bacteremia in children with SMA,^5, 9, 10^ but not CM.^10, 28^ A study in Malawian children with severe malaria reported a 43% increase in the risk of NTS bacteremia per 1g/dl reduction in hemoglobin levels.^9^ However, these observations have not been universal. A study in Mozambican children reported no clear-cut association between SMA and NTS bacteremia, although few children had NTS bacteremia (n=12).^13^ In agreement with the current study, previous studies have found no association between malaria and risk of NTS bacteremia,^4, 23^ although other studies have reported mixed findings with malaria both reducing and increasing risk of NTS bacteremia^29-32^. These differences might be explained by the prevalence of malarial anemia within the study populations or various other factors, including nutritional status. Taken together, our findings suggest that SMA, rather than other malarial phenotypes, underlies the association between malaria and NTS bacteremia. Indeed, severe anemia due to all causes was strongly associated with NTS bacteremia, even after excluding children with malaria parasitemia, in agreement with a previous study in Malawian children.^33^

A number of pathways may contribute to increased risk of NTS bacteremia in children with SMA including hemolysis, iron overload and upregulation of heme oxygenase-1 (Figure 1).^14^ Hepcidin may also influence risk of NTS bacteremia in SMA by controlling the availability of iron for bacterial growth.^18-20^ We observed that hepcidin levels and hepcidin/ferritin ratios were lower in hospitalized children with SMA compared to those with CM. In agreement, a study in Kenyan children found lower hepcidin levels in malaria patients with severe anemia compared to those with higher hemoglobin levels.^34^ In contrast, a study in Nigerian children found no difference in hepcidin levels between children with SMA and CM and higher hepcidin levels in uncomplicated compared to severe malaria.^35^ Our findings may be explained by the low hepcidin/ferritin ratio and higher sTfR levels in SMA compared to CM, indicating increased erythropoietic activity. Severe anemia negatively regulates hepcidin production through the action of erythroferrone,^36^ even in the presence of inflammation/infection^37, 38^ or sickle cell disease.^39^ Inflammation, as measured by ferritin and CRP, did not differ between the SMA and CM groups, although parasite density, known to correlate with hepcidin levels,^40^ was higher in CM. In agreement with previous studies,^41, 42^ we found higher hepcidin levels in children with severe malaria compared to those with asymptomatic parasitemia. This is likely to be due to increased inflammation in severe malaria, rather than the older age of the community-based children, since older children would be expected to have higher hepcidin levels than younger children.^26, 43^

Iron is an essential nutrient for bacterial growth and ex-vivo studies suggest that increased serum iron levels may stimulate the growth of various bacteria including *Salmonella* Typhimurium.^44, 45^ In mouse models, reduced hepcidin levels are associated with increased susceptibility to NTS infections,^18^ although little is known about the role of hepcidin during NTS and malaria infections in children. In the current study, children with SMA+NTS had lower hepcidin levels and hepcidin/ferritin ratios than those with NTS alone; although sTfR, CRP and ferritin levels did not differ between these groups. High hepcidin levels would be expected in children with NTS bacteremia since hepcidin is known to increase in response to inflammation and infection. A challenge infection study with *Salmonella enterica* Typhi in the United Kingdom identified higher hepcidin concentrations during acute infection.^46^ *In-vitro* and murine studies also show that *Salmonella* Typhimurium may directly or indirectly upregulate hepcidin expression and perturb iron regulatory pathways.^20^

Hepcidin concentrations may alter iron availability within the *Salmonella*-containing vacuole (SCV). Recent evidence indicates that hepcidin leads to increased degradation of ferroportin on the SCV, and limits the movement of iron into the SCV. ^21^ However, this conflicts with an earlier report that ferroportin transports iron out of the SCV,^47^ and these contradictions may be based on differences in experimental systems used.^48^ It also remains controversial whether iron accumulation in the SCV may promote bacterial growth by increasing iron availability,^19, 22^ or kill bacteria through the Fenton reaction.^21^ Low hepcidin levels in mice with severe hemolytic anemia were associated with increased susceptibility to *Salmonella* Typhimurium infection and hepcidin treatment improved survival.^18^ We hypothesize that low hepcidin levels in children with SMA, and non-malaria severe anemia, might contribute to NTS bacteremia by increasing iron availability in the SCV for NTS growth together with other mechanisms (Figure 1). Surprisingly, sTfR levels were elevated in children with NTS alone despite higher hemoglobin levels. It is not known whether NTS might induce transcription of transferrin receptors to increase transferrin iron acquisition, since transferrin receptors have been observed on the SCV during early phases of NTS endocytosis in murine models.^49^

To the best of our knowledge, this is the first study reporting hepcidin levels in children with NTS or with concomitant SMA and NTS bacteremia. The strengths of the study are that we utilized a very large 21-year dataset (n=75,034) with matching stored samples to identify and describe associations between severe malaria, NTS bacteremia and hepcidin in children. Our study also has important limitations. First, the study was observational, and as such, associations may be subject to unmeasured confounders and reverse causality. Second, we had few samples for children with SMA and NTS coinfection and no samples for those with CM and NTS coinfection since some samples were insufficient or missing, which may have introduced selection bias. Nonetheless, these are a unique sample set with accompanying clinical data collected over 21 years. Another limitation is that we did not measure additional parameters such as serum iron, transferrin saturation, and haptoglobin levels due to the volumes and availability of stored samples. Additionally, a few participants had sTfR concentrations above the cut-off values making it challenging to interpret findings from regression models for sTfR (Supplementary Table S3). Finally, our study was conducted in a single site. It is also possible that our study underestimated associations, considering the low sensitivity of blood cultures used to identify NTS. Nonetheless, this study complements the existing *in vitro* and animal data on the relationship between SMA and NTS bacteremia and provides preliminary evidence on the possible role of hepcidin in mediating this association.

In conclusion, SMA was associated with a strongly increased risk of NTS bacteremia in children and reduced hepcidin levels were observed in children with SMA and SMA+NTS. The question of whether ferroportin transports iron into or out of the SCV remains an active area of research,^21, 47^ and future findings may support our hypothesis or generate new ideas on how low hepcidin might mediate NTS susceptibility in children with SMA. Further studies are needed to understand the role of the hepcidin-ferroportin axis in susceptibility to NTS in human subjects, how hepcidin and iron disturbances might mediate susceptibility to bacteremia due to NTS or other organisms, and how *P*. *falciparum*, iron deficiency or other etiologies of severe anemia influence this relationship.

## Supplementary Material

Supplementary File

## Figures and Tables

**Table 1:** Factors associated with non-typhoidal *Salmonella* bacteremia in all hospitalized children (n=75,034)

Characteristic	NTS, n (%)	Hospital controls, n (%)	OR (95% CI) ^1^	P^1^
**Clinical features**				
Age, years (IQR)	1.07 (0.55, 1.90)	0.98 (0.18, 2.18)	1.01 (0.93, 1.08)	0.87
Sex, male	224/400 (56.0)	42,226/74,633 (56.6)	0.98 (0.80, 1.19)	0.82
Fever	267/369 (72.4)	37,886/61,979 (61.2)	1.66 (1.32, 2.09)	<0.0001
Diarrhea ^2^	126/400 (31.5)	14,187/74,615 (19.0)	1.96 (1.58, 2.42)	<0.0001
Vomiting	127/388 (32.7)	18,061/73,242 (24.7)	1.49 (1.20, 1.84)	0.0003
Severe pneumonia ^3^	111/400 (27.8)	21,068/74,610 (28.2)	0.98 (0.78, 1.22)	0.83
Very severe pneumonia ^4^	59/400 (14.8)	7,480/74,606 (10.0)	1.55 (1.18, 2.05)	0.002
Underweight ^5^	204/340 (60.0)	28,479/68,661 (41.5)	2.12 (1.70, 2.63)	<0.0001
Stunting ^6^	198/371 (53.4)	27,812/69,510 (40.0)	1.72 (1.40, 2.11)	<0.0001
Wasting ^7^	180/380 (47.4)	19,493/67,493 (28.9)	2.22 (1.81, 2.71)	<0.0001
**Laboratory characteristics**				
Malaria slide positive	93/400 (23.3)	16,370/74,632 (21.9)	1.08 (0.85, 1.36)	0.52
SMA ^8^	38/393 (9.7)	2,253/74,223 (3.0)	3.42 (2.44, 4.79)	<0.0001
Cerebral malaria ^8,9^	8/286 (2.7)	1,719/63,244 (2.7)	1.00 (0.50, 2.02)	0.99
Non-SMA malaria	48/400 (12.0)	13,708/74,632 (18.4)	0.61 (0.45, 0.82)	0.001
Sickle cell disease	14/400 (3.7)	1,115/74,608 (1.5)	2.39 (1.40, 4.09)	0.002
HIV positive ^10^	38/139 (27.3)	1,756/35,327 (5.0)	7.19 (4.94, 10.48)	<0.0001
Hb, g/dl (IQR)	7.4 (5.2, 9.4)	9.8 (8.1, 11.6)	1.28 (1.24, 1.32)	<0.0001
Hb <5 g/dl	89/400 (22.3)	4,660/74,615 (6.2)	4.30 (3.39, 5.45)	<0.0001
Hb 5-7 g/dl ^11^	86/311 (27.7)	6,575/69,974 (9.4)	3.69 (2.87, 4.73)	<0.0001
Hb 7-10 g/dl ^12^	147/225 (65.3)	27,867/63,399 (44.0)	2.40 (1.83, 3.16)	<0.0001
Severe anemia without malaria^13^	44/400 (11.0)	1,997/74,634 (2.7)	4.50 (3.28, 6.17)	<0.0001

Abbreviations: NTS, non-typhoidal *Salmonella;* n/N, number positive/number tested; OR, odds ratio; CI, confidence interval; SMA, severe malaria anemia; and CM, cerebral malaria; Hb, hemoglobin; and HIV, human immunodeficiency virus.

1 Odds ratios and P values were derived from univariable logistic regression models;

2 Passage of three or more loose or liquid stools within 24 hours;

3 History of cough or difficulty in breathing plus lower chest wall indrawing;

4 Cough or difficulty breathing plus either prostration, lethargy, hypoxia, loss of consciousness, or a history of convulsions;

5 Weight-for-age z-score < –2;

6 Height-for-age z-score <–2;

7 Weight-for-height z-score <–2 or mid-upper arm circumference <12.5 cm in children >6 months of age using WHO Child Growth Standards^50^;

8 Children with overlapping SMA and CM clinical syndromes were excluded from analysis;

9 Only children with Blantyre coma scale scores were included;

10 Data was available from February 2005 after routine HIV testing was introduced thus analyses included a limited number of children (n = 35,466);

11 Excludes children with hemoglobin levels <5 g/dl;

12 Excludes children with hemoglobin levels <7 g/dl;

13 Severe anemia (hemoglobin <5 g/dl) and no malaria parasites on blood film.

**Table 2:** Hepcidin and biomarkers of iron status and inflammation in a sub-study of hospitalized and community-based children

Biomarker	Group	n	Medians (IQR)	P^1^
Hepcidin, ng/ml	SMA and NTS coinfection	16	9.3 (4.7, 49.8)	Reference
	Severe malaria anemia	33	31.1 (5.5, 61.2)	0.43
	NTS bacteremia	33	105.8 (17.3, 233.3)	0.02
	Cerebral malaria	34	90.7 (38.7, 176.1)	0.004
	Asymptomatic malaria	49	6.5 (2.0, 13.1)	0.16
	Healthy controls	242	3.8 (1.2, 12.6)	0.01
Ferritin, μg/L	SMA and NTS coinfection	16	311.5 (241, 392)	Reference
	Severe malaria anemia	32	348.5 (296, 384)	0.55
	NTS bacteremia	29	356.0 (203, 397)	0.76
	Cerebral malaria	28	370.0 (359, 393)	0.23
	Asymptomatic malaria	48	30.5 (17.0, 53.0)	<0.0001
	Healthy controls	237	16.0 (8.0, 26.0)	<0.0001
Hepcidin/ferritin ratio^2^	SMA and NTS coinfection	16	0.03 (0.01, 0.22)	Reference
	Severe malaria anemia	32	0.10 (0.03, 0.19)	0.53
	NTS bacteremia	29	0.31 (0.06, 0.66)	0.007
	Cerebral malaria	28	0.24 (0.14, 0.69)	0.01
	Asymptomatic malaria	48	0.19 (0.09, 0.46)	0.01
	Healthy controls	232	0.27 (0.08, 0.66)	0.0006
sTfR, mg/L	SMA and NTS coinfection	16	48.1 (36.8, 66.9)	Reference
	Severe malaria anemia	33	43.3 (30.1, 61.3)	0.64
	NTS bacteremia	32	38.3 (30.9, 67.6)	0.50
	Cerebral malaria	33	31.2 (23.9, 45.5)	0.02
	Asymptomatic malaria	49	3.8 (2.7, 4.9)	<0.0001
	Healthy controls	239	3.6 (2.8, 4.8)	<0.0001
CRP, mg/L	SMA and NTS coinfection	16	117.5 (79.0, 145.0)	Reference
	Severe malaria anemia	33	120.0 (79.2, 152.7)	0.80
	NTS bacteremia	33	104.3 (35.1, 162.4)	0.67
	Cerebral malaria	33	120.9 (57.8, 150.9)	0.93
	Asymptomatic malaria	48	1.0 (0.3, 7.1)	<0.0001
	Healthy controls	237	0.3 (0.3, 2.0)	<0.0001

Abbreviations: IQR, interquartile range; SMA, severe malaria anemia; NTS, non-typhoidal *Salmonella*; CRP, C-reactive protein; sTfR, soluble transferrin receptor; and n/a, data not available.

1 P values were derived using pairwise Wilcoxon rank sum test.

2 Hepcidin/ferritin ratio was calculated by dividing hepcidin (ng/ml) by ferritin (μg/L).

## Data Availability

The data and analyses scripts underlying this article are available in Harvard Dataverse at https://doi.org/10.7910/DVN/KXZWN6 and applications for data access can be made through the Kilifi Data Governance Committee cgmrc@kemri-wellcome.org.
