# Supplementary material for: Hepcidin regulation in Kenyan children with severe malaria and non-typhoidal Salmonella bacteremia
Source: Haematologica. Author manuscript; Available in PMC 2022 Jul 5. (PMC9244826; doi:10.3324/haematol.2021.279316)
Supplement: Supplementary File [file EMS142309-supplement-Supplementary_File.pdf]

**Hepcidin regulation in Kenyan children with severe malaria and non-typhoidal *Salmonella* bacteremia**

Kelvin M. Abuga[1,2], John Muthii Muriuki[1], Sophie M. Uyoga[1], Kennedy Mwai[1,3], Johnstone Makale[1], Reagan M. Mogire[1,4], Alex W. Macharia[1,4], Shebe Mohammed[1], Esther Muthumbi[1], Salim Mwarumba[1], Neema Mturi[1], Philip Bejon[1,5], J. Anthony G. Scott[1,6], Manfred Nairz[7], Thomas N. Williams[1,5,8], and Sarah H. Atkinson[1,5,9]

**Authors' affiliations**

[1]Kenya Medical Research Institute (KEMRI) Center for Geographic Medicine Research, KEMRI-Wellcome Trust Research Programme, Kilifi, Kenya;

[2]Department of Public Health, School of Human and Health Sciences, Pwani University, Kilifi, Kenya;

[3]Epidemiology and Biostatistics Division, School of Public Health, University of the Witwatersrand, South Africa;

[4]Open University, KEMRI-Wellcome Trust Research Programme – Accredited Research Centre, Kilifi, Kenya;

[5]Centre for Tropical Medicine and Global Health, Nuffield Department of Clinical Medicine, University of Oxford, Oxford, UK;

[6]Department of Infectious Disease Epidemiology, London School of Hygiene and Tropical Medicine, London, UK;

[7]Department of Internal Medicine II, Medical University Innsbruck, Innsbruck, Austria;

[8]Department of Infectious Diseases and Institute of Global Health Innovation, Imperial College, London, UK; and

[9]Department of Paediatrics, University of Oxford, Oxford, UK

## Supplementary Methods

### *Ethical considerations*

Ethical approval was granted by the Scientific Ethics Review Unit of the Kenya Medical Research Institute (protocols KEMRI/SERU/CGMRC-C/155/3857 and KEMRI/SERU/CGMRC/046/3257). Individual written informed consent was provided by parents or guardians of study participants, or with a thumbprint if not literate with a signature from a literate witness.

### *Sickle cell diagnosis*

DNA was extracted retrospectively from frozen samples collected at admission by use of Qiagen DNA blood mini kits (Qiagen, Crawley, UK) and typed for sickle-cell disease by polymerase chain reaction (PCR) as previously described.[1, 2]

### *Iron and inflammatory biomarker assays*

We assayed hepcidin (Hepcidin-25 [human] EIA kit; Bachem), soluble transferrin receptor (sTfR; enzyme-linked immunosorbent assay; R&D systems), ferritin (micro-particle enzyme immunoassay, IMx [MEIA] ferritin assay, Abbott Laboratories), complete blood count (Beckman Coulter) and C-reactive protein (CRP, Dade Dimension particle enhanced turbidimetric immunoassay; Hitachi Corp.) according to manufacturers guidelines and as previously described.[3] Hepcidin/ferritin ratio was calculated by dividing hepcidin (ng/ml) by ferritin (µg/L).

### *Statistical analyses*

To examine factors associated with risk of NTS bacteremia, we used univariable logistic regression models, and a stepwise backward selection approach eliminating factors with $P>0.1$ after each step and retaining those with $P<0.05$ in the final multivariable logistic regression models. Variables defined by hemoglobin levels (including severe anemia, SMA, and severe anemia without malaria) were included in separate models for the multivariable analyses.Year of admission was included as a covariate in the final models. Wasting and stunting were dropped out of the final models because of collinearity with underweight. HIV status was not included in the final multivariable models due to many missing values, but remained a significant predictor of NTS bacteraemia in analyses restricted to children with HIV diagnosis (data not shown).

## Supplementary Tables

**Supplementary Table S1.** Sample selection for the hepcidin sub-study across the years

| | **1998** | **1999** | **2000** | **2001** | **2002** | **2003** | **2004** | **2005** | **2006** | **2007** | **2008** | **2009** | **2010** | **2011** | **Total** |
|---|---|---|---|---|---|---|---|---|---|---|---|---|---|---|---|
| SMA+NTS | 1 | 10 | 2 | 0 | 0 | 1 | 0 | 1 | 0 | 0 | 0 | 0 | 1 | 0 | **16** |
| SMA | 4 | 4 | 1 | 0 | 0 | 8 | 3 | 7 | 5 | 0 | 0 | 0 | 1 | 0 | **33** |
| NTS | 3 | 5 | 3 | 0 | 1 | 9 | 0 | 2 | 3 | 2 | 1 | 0 | 3 | 1 | **33** |
| CM | 1 | 0 | 0 | 1 | 1 | 9 | 5 | 0 | 10 | 7 | 0 | 0 | 0 | 0 | **34** |

Abbreviations: SMA, severe malarial anemia; NTS, non-typhoidal *Salmonella;* and CM, cerebral malaria.

**Supplementary Table S2.** Stepwise-selection multivariable logistic regression of factors associated with non-typhoidal *Salmonella* bacteremia in all hospitalized children (n=75,034)

| **Characteristic**[1] | **SMA-restricted model** | | **All severe anemia– restricted model** | | **Severe anemia without malaria – restricted model** | |
|---|---|---|---|---|---|---|
| | Adj OR (95% CI) [2] | Adj P [2] | Adj OR (95% CI) [2] | Adj P [2] | Adj OR (95% CI) [2] | Adj P [2] |
| SMA | 2.17 (1.44, 3.28) | 0.0002 | | | | |
| Severe anaemia (Hb <5 g/dl) | | | 3.15 (2.36, 4.20) | <0.0001 | | |
| Severe anemia without malaria | | | | | 4.03 (2.78, 5.84) | <0.0001 |
| Age, years | 0.88 (0.80, 0.97) | 0.009 | 0.87 (0.79, 0.96) | 0.004 | 0.88 (0.80, 0.97) | 0.009 |
| Fever (temperature >37.5ºC) | 1.89 (1.46, 2.45) | <0.0001 | 1.85 (1.43, 2.39) | <0.0001 | 1.85 (1.43, 2.39) | <0.0001 |
| Diarrhea [3] | 1.58 (1.22, 2.03) | 0.0004 | 1.65 (1.28, 2.11) | 0.0001 | 1.54 (1.20, 1.97) | 0.0007 |
| Very severe pneumonia [4] | 1.54 (1.12, 2.12) | 0.008 | 1.46 (1.06, 2.01) | 0.02 | 1.53 (1.12, 2.10) | 0.008 |
| Underweight [5] | 2.19 (1.74, 2.76) | <0.0001 | 2.06 (1.64, 2.59) | <0.0001 | 2.10 (1.67, 2.64) | <0.0001 |
| Sickle cell disease | 2.55 (1.33, 4.88) | 0.005 | 1.97 (1.03, 3.78) | 0.04 | | |

Abbreviations: OR, odds ratio; CI, confidence interval; SMA, severe malaria anemia. [1]Only factors with P<0.05 in the final models are shown. SMA, severe anaemia, and severe anemia without malaria were included in separate models. [2]Adjusted odds ratios and P-values from a stepwise backward selection logistic regression model retaining variables with P<0.1 in each step, and including variables with P<0.05 in the final models. Year of admission was also adjusted in the final models. HIV status was not included in the final multivariable models due to many missing values, but remained a significant predictor of NTS bacteraemia in analyses restricted to children with HIV diagnosis (adj. OR 5.07 [95% CI 3.14, 8.19]; P<0.0001 in the SMA model); [3]Passage of three or more loose or liquid stools within 24 hours; [4]Cough or difficulty breathing plus either prostration, lethargy, hypoxia, loss of consciousness, or a history of convulsions; [5]Weight-for-age z-score < –2 using WHO Child Growth Standards.

**Supplementary Table S3.** Demographic and clinical characteristics of children in the hepcidin sub-study with measurements of iron and inflammation

| **Characteristic** | **Hospitalized children** | | | | **Community children**[1] | |
|---|---|---|---|---|---|---|
| | SMA and NTS (%) | SMA (%) | NTS (%) | CM (%) | AM (%) | Healthy controls (%) |
| Age, months (IQR) [2] | 23.6 (11.4, 31.6) | 22.3 (16.6, 30.3) | 17.2 (5.5, 28.5) | 23.0 (13.6, 36.4) | 63.2 (40.1, 77.4) | 41.6 (21.7, 64.2) |
| Sex, male | 8/16 (50.0) | 15/33 (45.5) | 20/33 (60.6) | 13/34 (38.2) | 27/49 (55.1) | 134/242 (55.4) |
| Fever [3] | 7/16 (43.8) | 21/33 (63.6) | 18/32 (56.3) | 25/34 (73.5) | 3/21 (14.3) | 5/88 (5.7) |
| Hemoglobin | 3.2 (2.7, 4.2) | 4.0 (3.4, 4.4) | 7.3 (6.4, 9.8) | 7.5 (5.9, 8.2) | n/a | n/a |
| Vomiting | 7/16 (43.8) | 16/33 (18.2) | 9/33 (27.2) | 10/33 (30.3) | n/a | n/a |
| Wasting | 9/16 (56.3) | 8/33 (28.2) | 15/32 (45.5) | 8/32 (23.5) | n/a | n/a |
| Stunting | 9/16 (56.3) | 13/30 (43.3) | 14/29 (48.3) | 14/31 (45.2) | n/a | n/a |
| Underweight | 7/12 (58.3) | 11/32 (34.3) | 20/29 (69.0) | 16/34 (47.1) | n/a | n/a |
| Pallor [4] | 5/5 (100) | 24/25 (96.0) | 10/27 (37.0) | 20/33 (60.6) | n/a | n/a |
| Coma (BCS <3) | 0/3 (0) | 5/24 (20.8) | 1/22 (4.6) | 34/34 (100.0) | n/a | n/a |
| HIV status, positive [5] | 0/1 (0) | 2/9 (22.2) | 2/9 (18.2) | 0/17 (0) | n/a | n/a |
| Transfused | 14/16 (87.5) | 19/33 (57.6) | 3/33 (9.1) | 9/34 (26.5) | n/a | n/a |
| In-hospital mortality | 4/16 (25.0) | 3/33 (9.1) | 8/33 (24.2) | 4/34 (11.8) | n/a | n/a |

Abbreviations: SMA, severe malaria anemia; NTS, non-typhoidal *Salmonella*; CM, cerebral malaria; AM, asymptomatic malaria; IQR, interquartile range; BCS, Blantyre coma score; and HIV, human immunodeficiency virus. [1]Only age, gender and axillary temperature data were available for community children. [2] Medians and interquartile ranges are presented. [3]Temperature >37.5ºC. [4]Pallor was defined clinically. [5]HIV data was only available for children admitted between 2005-2019.

**Supplementary Table S4.** Geometric means and linear regression analyses of iron and/or inflammatory biomarkers by hospital groups.

| Biomarker | Group | n | Geometric means (95% CI) | Adj β (95% CI)[1] | Adj P[1] |
|---|---|---|---|---|---|
| Log-hepcidin, ng/ml | SMA and NTS | 16 | 11.7 (4.0, 34.5) | Reference | |
| | SMA | 33 | 21.4 (12.4, 36.8) | 0.47 (-0.73, 1.67) | 0.44 |
| | NTS | 33 | 48.7 (20.3, 116.4) | 1.99 (0.81, 3.26) | 0.001 |
| | CM | 34 | 62.9 (37.2, 106.4) | 1.52 (0.13, 2.92) | 0.03 |
| Log-ferritin, µg/L | SMA and NTS | 16 | 287.0 (233.4, 352.9) | Reference | |
| | SMA | 32 | 329.3 (280.7, 386.4) | 0.26 (-0.08, 0.61) | 0.14 |
| | NTS | 29 | 268.6 (193.8, 372.3) | 0.22 (-0.12, 0.56) | 0.20 |
| | CM | 28 | 305.5 (226.8, 411.5) | 0.37 (-0.03, 0.77) | 0.07 |
| Log-hepcidin/ferritin | SMA and NTS | 16 | 0.04 (0.01, 0.12) | Reference | |
| | SMA | 32 | 0.07 (0.04, 0.12) | 0.15 (-1.04, 1.35) | 0.80 |
| | NTS | 29 | 0.18 (0.08, 0.40) | 1.56 (0.38, 2.74) | 0.01 |
| | CM | 28 | 0.20 (0.11, 0.38) | 1.03 (-0.37, 2.43) | 0.15 |
| Log-sTfR, mg/L[2] | SMA and NTS | 16 | 46.2 (36.5, 58.6) | Reference | |
| | SMA | 33 | 42.8 (35.7, 51.4) | -0.19 (-0.52, 0.14) | 0.25 |
| | NTS | 32 | 40.6 (32.6, 50.4) | -0.23 (-0.55, 0.09) | 0.16 |
| | CM | 33 | 33.9 (29.8, 40.0) | -0.40 (-0.78, -0.01) | 0.04 |
| Log-CRP, mg/L | SMA and NTS | 16 | 98.4 (72.9, 132.9) | Reference | |
| | SMA | 33 | 96.7 (76.6, 122.0) | -0.04 (-0.72, 0.63) | 0.90 |
| | NTS | 30 | 77.2 (51.7, 115.1) | -0.26 (-0.92, 0.39) | 0.43 |
| | CM | 33 | 76.2 (51.6, 112.7) | -0.34 (-1.12, 0.44) | 0.39 |
| Log-parasite density, parasites/µl | SMA and NTS | 16 | $9.1 \times 10^3$ ($3.0 \times 10^3$, $2.8 \times 10^4$) | Reference | |
| | SMA | 33 | $5.1 \times 10^4$ ($2.4 \times 10^4$, $1.1 \times 10^4$) | 1.49 (0.09, 2.90) | 0.04 |
| | CM | 33 | $1.9 \times 10^5$ ($1.1 \times 10^5$, $3.2 \times 10^5$) | 2.87 (1.12, 4.60) | 0.002 |

Abbreviations: SMA, severe malaria anaemia; NTS, non-typhoidal *Salmonella* bacteremia; sTfR, soluble transferrin receptors; CRP, and C-reactive protein. [1]Adjusted coefficients (adj β) and P-values were derived from a linear regression model adjusting for inflammation (log-CRP) and year of admission; [2]Twelve sTfR values were above the upper limit of the assay (>84 mg/L) and were recorded as 84 mg/L for this analysis. These values were distributed across the groups as follows: SMA and NTS (2), SMA (5), NTS (2) and CM (3).

**Supplementary Table S5.** Correlation of hepcidin with iron and inflammatory biomarkers in hospitalized children

| | All groups | | SMA and NTS | | SMA | | NTS | | CM | |
|---|---|---|---|---|---|---|---|---|---|---|
| **Variable** | r | P | r | P | r | P | r | P | r | P |
| Ferritin, µg/L | 0.38 | <0.0001 | 0.10 | 0.69 | 0.37 | 0.04 | 0.58 | 0.001 | 0.21 | 0.28 |
| sTfR, mg/L [1] | -0.37 | <0.0001 | -0.08 | 0.77 | -0.43 | 0.01 | -0.34 | 0.06 | -0.21 | 0.24 |
| CRP, mg/L | 0.31 | 0.0007 | 0.24 | 0.37 | 0.63 | 0.0001 | 0.44 | 0.01 | 0.02 | 0.91 |
| Hemoglobin, g/dL | 0.37 | <0.0001 | 0.32 | 0.23 | 0.28 | 0.11 | -0.07 | 0.70 | 0.20 | 0.25 |
| Parasite density, parasites/µl | 0.44 | <0.0001 | -0.08 | 0.77 | 0.53 | 0.001 | | | 0.29 | 0.09 |

Abbreviations: SMA, severe malaria anemia; NTS, non-typhoidal *Salmonella* bacteremia; CM, cerebral malaria; sTfR, soluble transferrin receptors; CRP, C-reactive protein; and r, pairwise non-parametric Spearman's correlation coefficients. [1]Twelve sTfR values were above the upper limit of the assay (>84 mg/L) and were recorded as 84 mg/L for this analysis. These values were distributed across the groups as follows: SMA and NTS (2), SMA (5), NTS (2) and CM (3).

**Supplementary Figure S1**

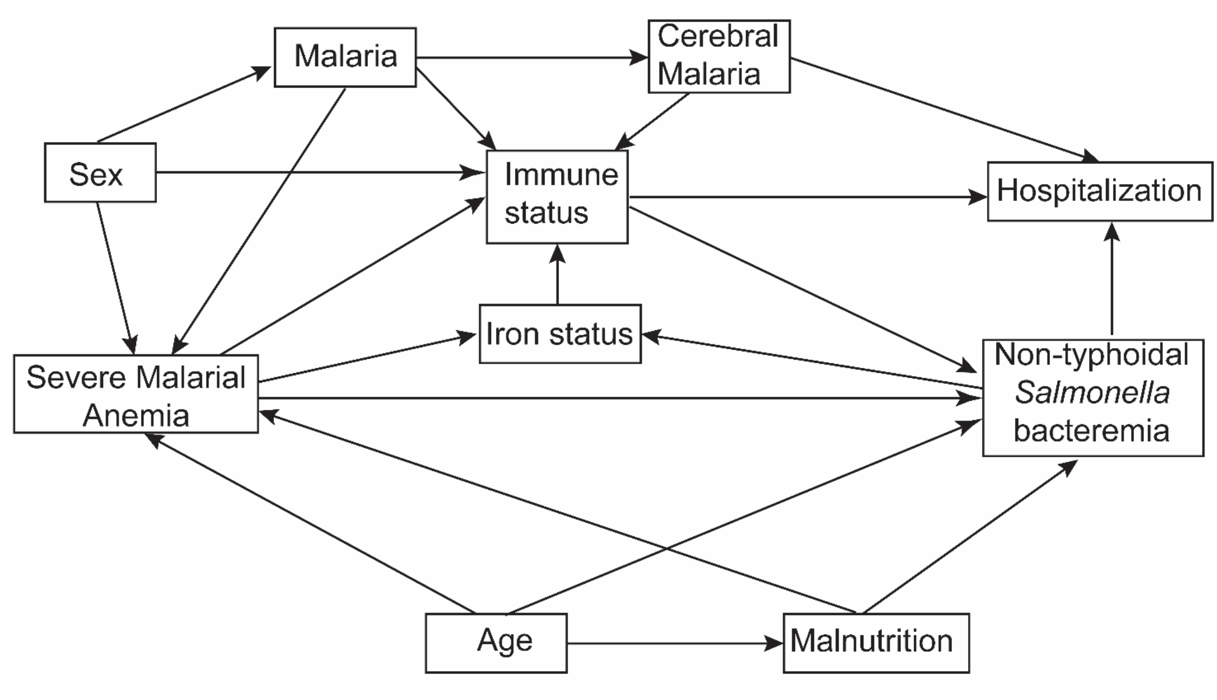


**Supplementary Figure S1**. Directed acyclic graph for the causal pathways between malaria and non-typhoidal *Salmonella*.

**Supplementary Figure S2**

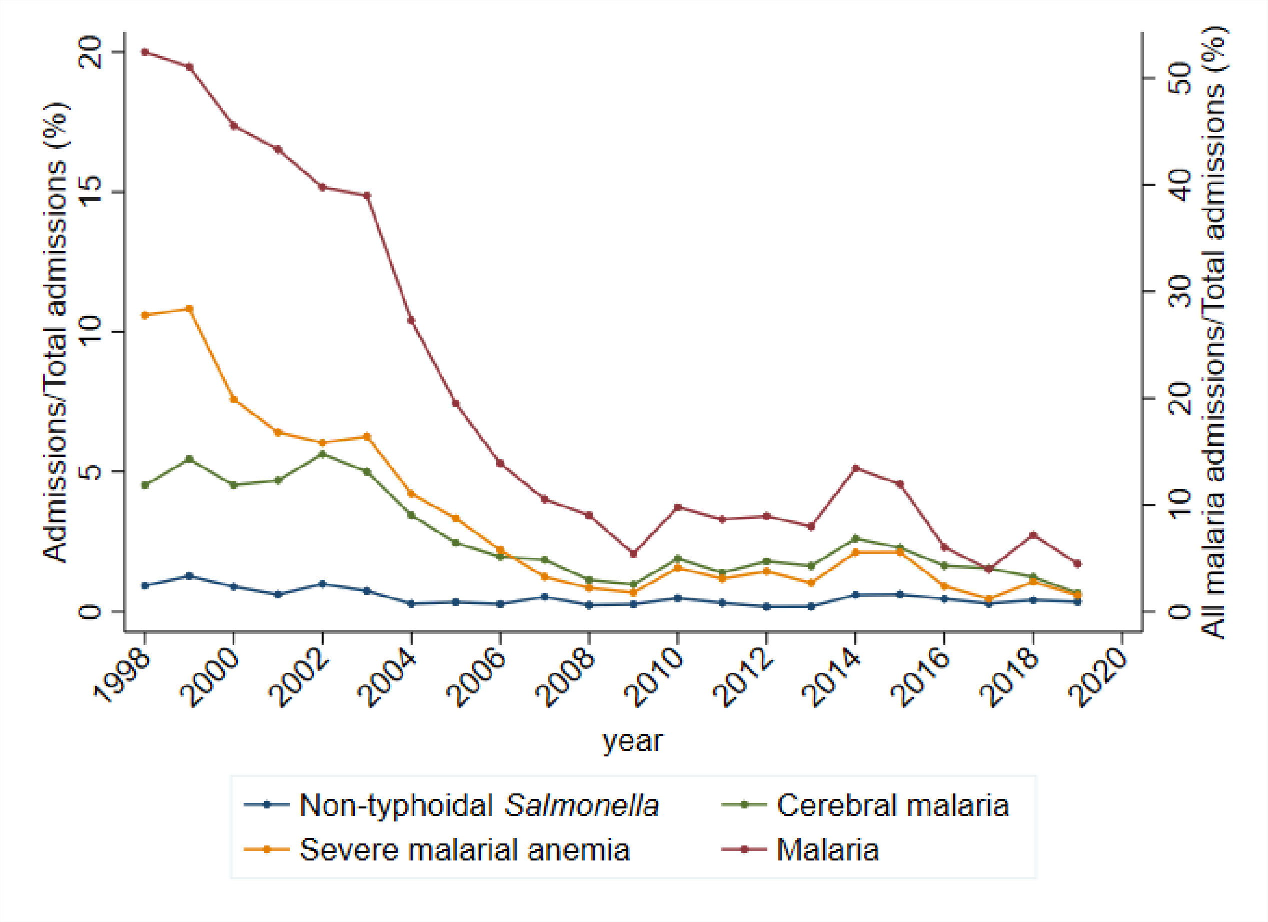


**Supplementary Figure S2.** Proportions of admissions (in percentage) due to non-typhoidal *Salmonella*, malaria, cerebral malaria, and severe malarial anaemia between August 1998 and October 2019.

**Supplementary Figure S3**

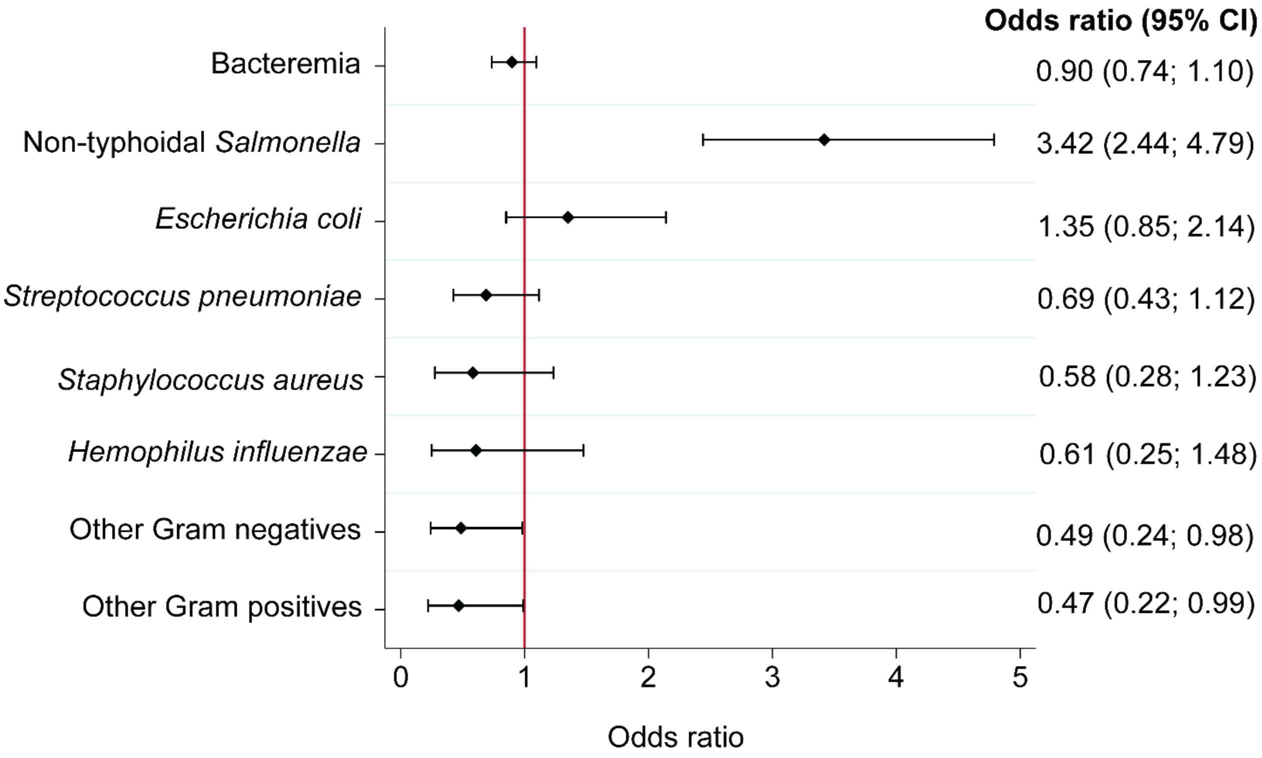


**Supplementary Figure S3.** Forest plot showing association between SMA and the risk of various organisms causing bacteraemia in hospitalized children. Odds ratios were derived from univariable logistic regression models (n=75,034).

References

1. Williams TN, Uyoga S, Macharia A, et al. Bacteraemia in Kenyan children with sickle-cell anaemia: a retrospective cohort and case-control study. Lancet. 2009;374(9698):1364-1370.

2. Macharia AW, Mochamah G, Uyoga S, et al. The clinical epidemiology of sickle cell anemia In Africa. Am J Hematol. 2018;93(3):363-370.

3. Atkinson SH, Uyoga SM, Armitage AE, et al. Malaria and Age Variably but Critically Control Hepcidin Throughout Childhood in Kenya. EBioMedicine. 2015;2(10):1478-1486.
